# Effect of Postharvest Application of Aloe Vera Gel on Shelf Life, Activities of Anti-Oxidative Enzymes, and Quality of ‘Gola’ Guava Fruit

**DOI:** 10.3390/foods9101361

**Published:** 2020-09-25

**Authors:** Muhammad Adil Rehman, Muhammad Rafique Asi, Amjad Hameed, Leslie D. Bourquin

**Affiliations:** 1 Nuclear Institute for Agriculture and Biology College, Pakistan Institute of Engineering and Applied Sciences (NIAB-C, PIEAS), Jhang Road, Faisalabad 38000, Pakistan; asimuhammad@yahoo.co.uk (M.R.A.); amjad46pk@yahoo.com (A.H.); 2 Department of Food Science and Nutrition, Michigan State University, East Lansing, MI 48824-1223, USA

**Keywords:** edible coating, aloe vera gel, shelf life, guava, postharvest, antioxidant, high-performance liquid chromatography

## Abstract

Guava is an important climacteric fruits in terms of taste and aroma, which contains various vital nutrients such as minerals, carotenoids, ascorbic acid, and polyphenols. At ambient conditions, it exhibits a short shelf life, which makes it difficult for marketing and subsequent storage. Therefore, it is necessary to develop procedures to extend its shelf life and conserve quality. For this purpose, an aloe vera (AV) gel coating was assessed for its potential to enhance the shelf life of guava fruits. Guava fruits coated with AV gels (0, 20, 40, 60 and 80%, *v*/*v*) were evaluated for postharvest shelf life extension, changes in quality attributes, anti-oxidative activities, and flavonoid content when stored at ambient conditions (23 ± 2 °C and 70–75% relative humidity) for 12 days. The AV gel-treated fruits showed reduced increments in total sugar, malondialdehyde, and total carotene contents compared to untreated controls. AV gel-treated fruits exhibited higher contents of ascorbic acid, flavonoids (quercetin and rutin), and total phenolics in comparison to control fruits. Moreover, AV gel-treated fruits displayed greater activities of superoxide dismutase, catalase, and ascorbate peroxidase, along with higher antioxidant capacity and higher levels of total soluble solids, than untreated fruits. These results demonstrate that AV gel coating, especially at high concentrations, can be considered an eco-friendly and non-chemical substitute treatment for maintaining the postharvest quality of guava fruit.

## 1. Introduction

The guava (*Psidium guajava* L.) fruit is highly palatable and a rich source of vitamin C. Pakistan, Brazil, South Africa, Mexico, India, Venezuela, Egypt, and Columbia are major guava-producing countries. In Pakistan, the total area of guava cultivation is 62,300 ha, producing 512,300 t of fruits per annum. After citrus, mango, and apple, guava ranks fourth in Pakistan in terms of area and production [1]. Nutritionally, guava fruit is a rich source of ascorbic acid (0.5–0.30 mg/g fresh weight), whose content is three to six times higher than in oranges [2]. The nutritional significance of guava is due to its high dietary value, being a source of carotenoids, polyphenols [3], pectin (0.5–1.8%), dietary fiber, vitamin A, phosphorus, niacin, calcium, iron, riboflavin, thiamine [4].

The guava fruit undergoes many physiological changes during and after harvest, which accelerate ripening processes and, consequently, promote deterioration. High temperatures, low levels of atmospheric humidity, and physical injuries accelerate the deterioration of guava fruit after harvest. In order to combat these losses, the application of an edible coating is an emerging solution that is inexpensive, easily available, and effective in prolonging the shelf life of food commodities [5]. Coatings which are made from natural ingredients are generally safe for human consumption and are also environmentally friendly [6]. On guava fruits, chitosan coating has been used, and it was observed that this coating solution effectively enhanced the shelf life of guava fruits by controlling disease and maintaining guava fruit quality parameters during storing at ambient temperature [7].

Aloe vera (AV) is a short-stemmed succulent plant species belonging to the Asphodeleceae (*Liliaceae*) family. It commonly grows in arid regions of most continents (Europe, Asia, Africa, and America). The AV plant stem has a high tendency to survive in severe circumstances because it can retain moisture in warm and dry climates [8]. For centuries, many traditions and cultures have used AV for medicinal purposes. AV gel inhibits the growth of various pathogenic and foodborne spoilage organisms including *Staphylococcus aureus*, *Salmonella*, *Streptococcus*, *Escherichia coli*, *Aspergillus niger*, *Candida*, etc. [9]. Moreover, AV gel coatings reduced O_2_ consumption and CO_2_ production, thereby preventing anaerobic conditions [10]. Polysaccharides in AV gel act as a barrier to moisture and O_2_, resulting in a slow respiration rate and conserving fruit quality [11]. Recently, AV gel coating for fruits has been studied by several researchers, who have reported that the AV gel creates a modified atmosphere of internal gases, which reduces moisture loss, respiration rate, oxidative browning, softening of tissues, and proliferation of microorganisms in fruits, including nectarines [12], sweet cherries [13], and table grapes [14]. AV gel coatings in combination with additives also maintain the color and firmness in fresh-cut slices of apple [15]. The combination of AV with salicylic acid was more effective in maintaining quality and decreasing microbial load compared to the individual substances in orange (*Citrus sinensis* L) fruit [16]. The gel from the other cultivar of aloe vera (*Aloe arborescens*) has also been used for postharvest application on various fruits. Recently, it was used as an edible coating for strawberries, reducing total soluble solids (TSS) increasing the concentrations of total phenolics compounds (TPC) and ascorbic acid (AsA), and improving the antioxidant activity in comparison to non-coated fruits [17].

Calcium salts have been widely used in the food industry, especially in the fruit and vegetable industry, as a preservative and firming agent. It plays a vital role in maintaining the cell wall structure [3]. Papaya and peach fruits treated with CaCl_2_ showed greater firmness and better shelf life than untreated fruits [18,19]. In ready-to-eat products, CaCl_2_ also reduces the ripening rate and color loss without changing the qualities of the products and maintaining a high content of TSS [20]. Additionally, the presence of AsA or its derivatives (0.5–4% (*w*/*v*) in fruit has been reported in many studies. AsA has great anti-browning and antimicrobial effects and is used as an additive in the formulation of edible coatings [21]. Many authors have described the effects of AsA on antimicrobial activity in whole and fresh-cut fruits such as apple [22], jackfruit [23], and papaya [21].

The present research was planned to assess the potential of an AV gel to enhance the postharvest quality and shelf life of guava fruits. The antioxidant profiles of AV gel-coated fruits were also analyzed to understand the potential mechanisms of quality and shelf life improvement.

## 2. Materials and Methods

### 2.1. Guava Fruits

Fine-quality guava fruits of the commercial cultivar “Gola” at full green color and firm stage were purchased from local farms (Shahzad Cheema Agri, Farm, Saloni Jhal) located in Faisalabad, Pakistan. Fruits without any sign of injury, free from insects, pests, and diseases, and of uniform size, shape, and color were selected. All fruits were washed before application of the AV gel.

### 2.2. Preparation of the AV Gel

Plants of aloe vera (*Aloe barbadensis* Mill) were grown in clay pots (5 kg soil) in a net house at Nuclear institute for agriculture and biology college (NIAB-C), Faisalabad, Pakistan. At maturity (180 days old), the leaves were cut with a sharp knife and brought to the Food Toxicology Laboratory, NIAB. The leaves (average weight, 415 g) were washed with tap water and shade-dried, and the gel was separated and blended to obtain a homogeneous mixture. Glycerol, citric acid, carboxymethyl cellulose (CMC), and calcium chloride (all purchased locally) were used for gel preparation. The pH of the gel was standardized to 3.75 with citric acid (5%), pasteurized at 90 °C for 15 min, cooled, and filtered using a muslin cloth. AV gel coating solutions (0 or control, 20, 40, 60, and 80%) were made up to the volume of 1 L by adding distilled water. AsA (4% *w*/*v*) as an antimicrobial agent, CaCl_2_ (3% *w*/*v*) as a firming agent, glycerol (1% *v*/*v*) as a plasticizer, and CMC (3% *w*/*v*) as a thickening agent were added to each solution. All the ingredients were homogenized to smooth mixtures of coating gel solutions. Tween 20, 0.03% (*v*/*v*), was added to the gel for adhesive purposes.

### 2.3. Treatment of Guava Fruit

A total of five treatments (0 or control, 20, 40, 60, and 80% AV gel) were used in this study. Fruits were treated with AV gel solutions in triplicate. All fruits were dipped for 3 min in AV gel solutions, dried at room temperature, packed in sintered polyurethane plastic baskets (30 cm length, 23 cm width, and 8 cm height,) and stored up to 12 days at 23 ± 2 °C and 70–75% relative humidity for visual observations and biochemical analyses. Data on quality parameters and shelf life were recorded at 3-day intervals.

### 2.4. Determination of TSS and Fruit Weight Loss

TSS were determined by using a digital hand refractometer (PAL-1, Atago, Tokyo, Japan) and expressed as °Brix [24]. The readings were standardized at room temperature (25 °C). The percent loss in weight was calculated by measuring the fruits’ weights using a digital balance. The first reading was taken of each dried fruit after dipping in the respective treatments before storage, and the second reading was taken at end of each sampling day [25].

### 2.5. Determination of Total Chlorophyll Content

Total chlorophyll content was measured by a spectrophotometer (U2800, HITACHI, Ibaraki, Japan) at 663 and 644 nm [7].

### 2.6. Determination of Total Sugar (TS), Reducing Sugar (RS), and Non-Reducing Sugar (NRS)

The total and reducing sugar contents of the fruits were determined by following a previously described method [26]. Non-reducing sugar contents were calculated by the difference between total sugar and reducing sugar.

### 2.7. Determination of Lipid Peroxidation (Malondialdehyde, MDA) Content

Lipid peroxidation level in the guava fruits treated with the AV gel formulations and in the control fruits was measured by the reaction of thiobarbituric acid (TBA) and trichloroacetic acid [27]. Absorbances were measured at 532 and 600 nm.

### 2.8. Total Phenolics Compounds (TPC)

The Folin–Ciocalteu (F–C) colorimetric method was used for the determination of TPC in guava juice [28]. The TPC concentrations were expressed as µM/g.

### 2.9. Determination of Total Carotenoid Content (TCC), AsA, and Total Antioxidant Capacity (TAC)

The TCC of all samples was determined spectrophotometrically on the basis of their chlorophyll content after measuring the absorbance (OD) at 663, 645, and 480 nm. The TCC was calculated by using the following formulas [29]:

$$\mathrm{Chlorophyll}\;A\;(\mathrm{mg}/g)=12.7\;\left(OD\;663\right)-2.69\;\left(OD\;645\right)\;\mathrm{Chlorophyll}\;B\;(\mathrm{mg}/g)=22.9\;\left(OD\;645\right)-2.69\;\left(OD\;663\right)\;\mathrm{Carotenoids}\;(\mathrm{mg}/g)=\frac{1000\;\left(OD\;480\right)-3.27\;\left(chlorophyll\;A\right)-104\;\left(chlorophyll\;B\right)}{227}$$

AsA content was measured by adding dichloroindophenol (DCIP) and metaphosphoric acid to the samples; after dilution with distilled water and thorough mixing, absorbance was measured at 520 nm [30]. The TAC of juice samples was determined by using the 2, 2’-Azino-Bis-3-Ethylbenzothiazoline-6-Sulfonic Acid (ABTS) method [31].

### 2.10. Determination of Total Flavonoids (TF)

TF were determined by diluting the samples with aluminum chloride (AlCl_3_) and potassium acetate (CH_3_CO_2_K) [32]. A linear equation was used to determine the total flavonoids. Quercetin and rutin were selected as standards (concentration range 0.005–0.1 mg/mL), and total flavonoids were expressed as mg/g of fruits extract.

### 2.11. Determination of Quercetin and Rutin by HPLC

#### 2.11.1. Sample Preparation

Guava juice samples were prepared and analyzed to determine the concentrations of rutin and quercetin. Extraction of flavonoids from guava juice samples for HPLC analysis was carried out by taking samples of guava juice (10 mL) in 50 mL Eppendorf tubes; 25 mL of 100% methanol was added to each tube [33]. The samples were sonicated for 20 min, then centrifuged at 10,000 rpm for 10 min at 25 °C. After centrifugation, the supernatants were collected, and methanol (5 mL) was added to the residues, which were centrifuged again at 10,000 rpm; the supernatants were again collected. The combined supernatants were filtered using Whatman No. 42 filter paper, and 5 g of anhydrous sodium sulfate was added to each supernatant to remove excess water.

#### 2.11.2. HPLC System and Conditions

Qualitative and quantitative determination of quercetin and rutin was conducted using HPLC (LC-10A, Shimadzu, Kyoto, Japan). Two solvents (A: 3% trifluoroacetic acid (TFA) and B: acetonitrile and methanol (80:20 *v*/*v*) were prepared. An equal mixture of these solvents (50:50 *v*/*v*) was used for the separation of flavonoids in isocratic mode with the following parameters:

HPLC system: LC-10 A, with a UV-Vis detector (SPD 10-A (λ 360 nm); flow rate: 0.8 mL/min; pressure: 67 kgf/cm^2^; injection volume: 20 µL; column temperature: 30 °C (CTO 10-A); analytical column: C18 (250 × 4.6 mm, 5 µm particle size, Supelco, Bellefonte, PA, USA); delivery pump: LC-10AS; system controller: SCL-10A; acquisition software: CLASS LC-10. The determination of individual flavonoids was made by comparing the retention times and peak areas to those of certified reference standards (quercetin, rutin, myricetin, and kaempferol).

#### 2.11.3. Standard Solution Preparation

Flavonoids (rutin and quercetin) were purchased from Sigma-Aldrich, USA. Different concentrations of standard were prepared in 100% methanol. A series of solutions (0, 10, 20, 50, 100, 200 and 300 µg/mL) was prepared to determine the limit of detection (LOD) and linearity of detector (response).

### 2.12. Superoxide Dismutase (SOD), Catalase (CAT), Ascorbate Peroxidase (APX)

Activities of SOD, CAT, and APX were assayed in the outer skin of the fruits. Fruit samples were homogenized in 2 mL potassium phosphate buffer (pH 7.0). Sample homogenization, extraction of enzymes, and centrifugation at 10,000 rpm for 10 min were the steps involved in performing the enzymes assay. After centrifugation, the collected supernatants were used for enzyme activity assays. All the steps were executed at 4 °C [34].

### 2.13. Statistical Analysis

Analysis of variance was used to analyze the data. All the parameters were assessed at the 5% significance level by using Tukey’s multiple comparison test by and the SAS 9.4 software. The complete experiment was conducted by factorial layout. The two factors were coating concentrations and storage days. Results are presented as mean ± SD. GraphPad Prism 8 (GraphPad Software, San Diego, CA, USA, http://www.graphpad.com) was used.

## 3. Results and Discussion

### 3.1. TSS and Fruit Weight Loss

**Figure 1:**
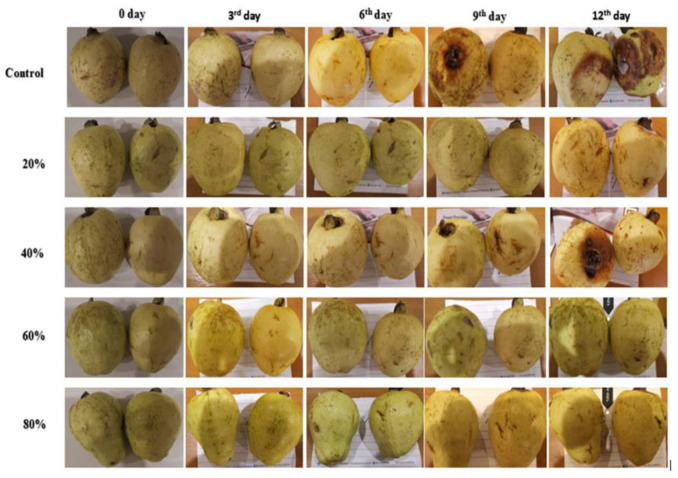
Effect of aloe vera (AV) gel coating on the storage life of guava fruits from the start until the end of the analysis on the 12th day.

**Figure 2:**
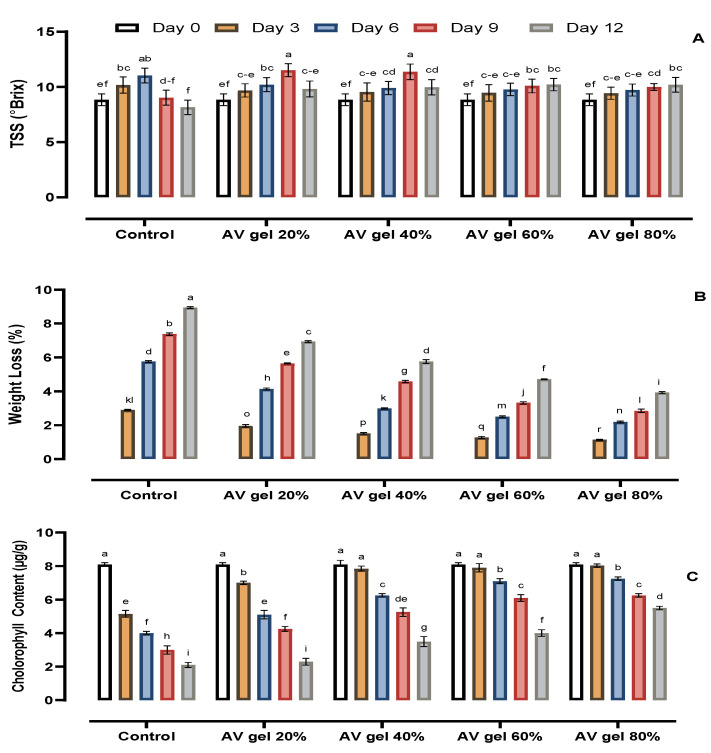
Result of AV gel coating on total soluble solids (TSS). (**A**), physiological weight loss (**B**), and chlorophyll content (**C**) in guava fruit. Standard error shown by vertical bars; each value represents the mean of three replicates. Different letters on the top of the bars indicate significant differences (*p* ≤ 0.05).

The effect of AV gel coating treatments on the guava fruits throughout the storage period of 12 days is visible in Figure 1. The TSS content in treated guava fruits increased gradually before decreasing until the 12th day of storage (Figure 2A). In treatments with AV gel 60% and AV gel 80%, a gradual increase in TSS was observed until the end of the trial, which indicates that the treatments with the AV gels had slowed the respiration rate of guava fruits during the storage period. Similar findings were reported previously, indicating a link between TSS and respiration rate [35]. The maximum increase in TSS content (11.03) was found in the control on the 6th day; the TSS value then decreased to 8.15 on the 12th day. In the case of treatments with AV gel 20% and AV gel 40%, the highest TSS content was observed on the 9th day (11.52 and 11.37, respectively); it then decreased to 9.82 and 9.97 at the end of the experiment. Treatments with AV gel 60% and AV gel 80% showed a steady increase of TSS from the beginning until the end of the study, with values from 8.84 to 10.21 and 8.84 to 10.19, respectively. TSS represents the carbohydrate content, and increased TSS could indicate cell wall deterioration. During storage, the TSS increased in both control and treated fruits (Figure 2A). The initial increase in TSS might be due to hydrolysis of starch into sugar; the subsequent declines in TSS could be due to the metabolism of sugars into organic acids and to a decreased respiration rate [36]. Furthermore, it has been reported that a lower TSS may be responsible for the phenomenon of hydrolysis of carbohydrates into sugar [37].

Weight loss was found for both control and treated fruits during the storage period. Coating of guava fruits with AV gel was effective in creating a physical barrier to moisture loss; hence, reduced weight loss was observed for the treated fruits (0 to 8.95%) than for the non-treated fruits (0 to 3.93%; Figure 2B). All the AV gel treatments were effective, but the smallest weight loss (3.93 on the 12th day of storage) was observed in fruits treated with the 80% AV gel. Weight loss in fruits and vegetables is an indication of water loss and increases due to desiccation and metabolic activities such as respiration and transpiration [38]. Previously, it was reported that loss of water from papaya was reduced by coating with chitosan [24]. Apart from guava fruit, AV gel coatings have been effective in controlling weight loss in other commodities. For instance, AV gel treatment reduced the weight loss and increased the shelf life of sapodilla [35] and cherry fruits [39]. Aloe vera gel also turned out to be appropriate for the extension of the shelf life of grapes at 20% concentration at low temperature storage by reducing moisture loss [40]. The probable reason for the reduced weight loss of AV gel-coated fruits is the inhibition of desiccation.

### 3.2. Total Chlorophyll Content

Guava fruits changed color from green to yellow during the storage period. The change in color was quantified by measuring the chlorophyll content of the fruits’ peel. AV gel treatments and storage time significantly influenced the chlorophyll content of guava fruits. The chlorophyll content decreased in AV gel-treated fruits as well as in control fruits, but the reduction rate was lower in treated fruits compared to the controls (Figure 2C). Fruit coated with AV gel 80% retained the peel color longer (8.1 to 5.5 µg/g) than the fruits undergoing the other treatments. The decline in chlorophyll content was extensive in control fruits and in fruits treated with AV gel 20%, which showed a decrease in chlorophyll content from 8.1 to 2.1 µg/g on the 12th day of storage. Guava fruits treated with AV gel 40% and AV gel 60% exhibited a smaller chlorophyll content reduction than the controls, with chlorophyll ranging from 8.1 to 3.5 µg/g and 8.1 to 4.0 µg/g, respectively, during the storage period. The AV gel delayed fruits’ senescence and ripening, which normally results in reduced fruit firmness and color change. It was also reported that guava fruits coated with chitosan exhibited reduced chlorophyll loss during storage [7]. The retardation of color development in papaya fruits treated with 2.0% of chitosan was attributed to a lower rate of ethylene production and a slower respiration, which led to a modified atmosphere for the fruit [24].

### 3.3. TS, RS, and NRS

**Figure 3:**
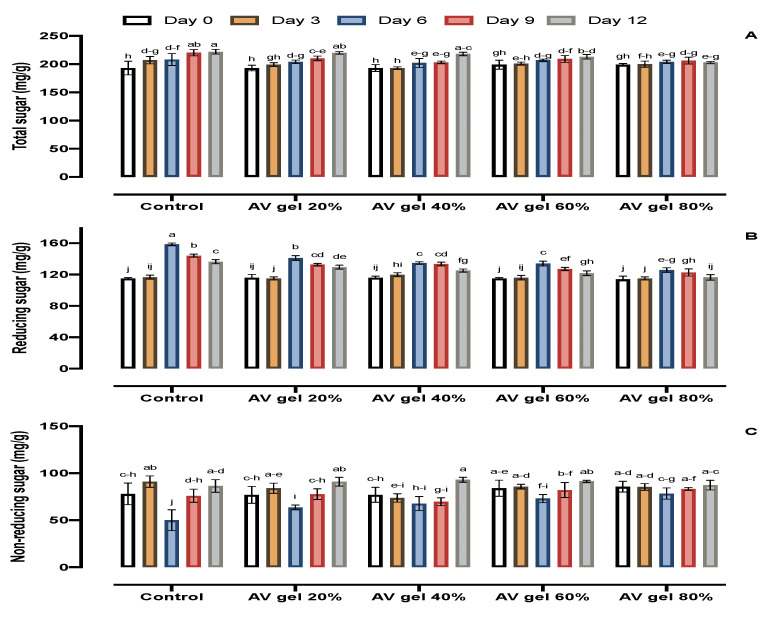
Effects of AV gel coating on total sugars (**A**), reducing sugars (**B**), and non-reducing sugars (**C**) in guava fruit. Standard error shown by vertical bars; each value is the mean of three replicates. Different letters on the top of bars indicate significant differences (*p* ≤ 0.05).

The effect of storage periods and AV gel treatments on reducing, non-reducing, and total sugars was significant. Regardless of the treatments, the total sugar content in guava fruits increased throughout the storage period (Figure 3A) but remained almost constant in fruits treated with the AV gel 80% (199 to 203 mg/g). The highest increment for this parameter was recorded in control fruits and in fruits treated with AV gel 20% (199 to 222 mg/g and 199 to 220 mg/g, respectively). Treatment with AV gels 40% and 60% attenuated the increase in TS during storage, with TS concentration increasing from 199 to 218 mg/g and 199 to 213 mg/g for these treatments, respectively. Overall, the sugar concentration of guava decreased reaching a peak on the 6th day of storage, then decreased gradually until the 12th day for all treatments (Figure 3B). After 12 days of storage, the reducing sugar concentration was significantly lower in guava fruits coated with AV gel 80% (114 to 116 mg/g) compared with control fruits (115 to 136 mg/g) (Figure 3B). Non-reducing sugars were initially increased on the 3rd day, showed a reduction on 6th day, increased again on 9th day, and then continued to increase until the 12th day, regardless of the treatment (Figure 3C). Up to the third day, the non-reducing sugar content was higher in control (91 mg/g) fruits, and on the nineth day, it declined (50 mg/g), then increasing (up to 86 mg/g) at end of the storage period. The maximum increase was observed in fruits treated with AV gel 40% and corresponded to 93 mg/g on the 12th day. However, the AV gel 80% retained the level of NRS from 0 day to the 12th day (85 to 87 mg/g), which indicated that guava fruits treated with AV gel 80% exhibited the best properties. Sugars concentration increases with ripening and senescence of fruits. Similarly, the conversion of starch may also contribute to increasing sugar content. Coating inhibits ripening, subsequent senescence, and conversion of starch into sugars [41]. Our results are in accordance with previous findings in guava obtained in China [42]. A similar conclusion regarding total sugar, reducing sugar, and non-reducing sugar was achieved using CaCl_2_ treatments during storage [36] of guava. Guava fruits coated with the 80% AV gel displayed a lower increase in sugar content, which may be due to the gel’s ability to slow down the conversion of starch into sugars.

### 3.4. Lipid Peroxidation (MDA) Content

**Figure 4:**
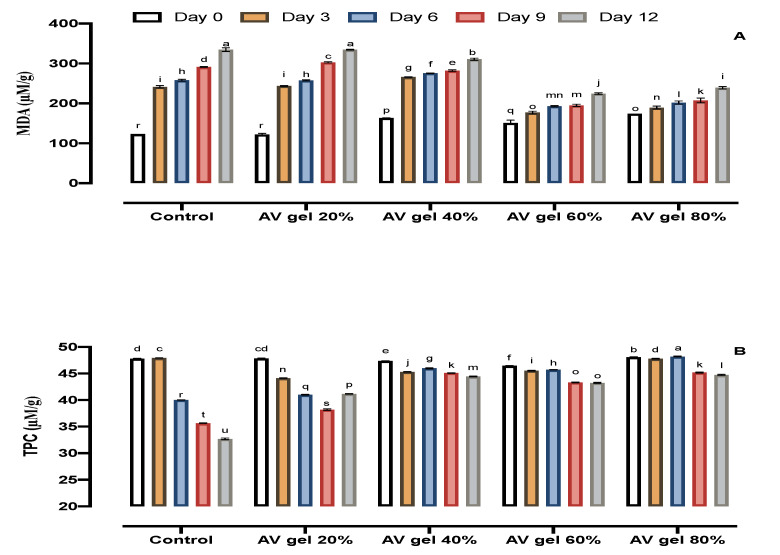
Effects of AV gel coating on malondialdehyde (MDA) production (**A**) and total phenolics compounds (TPC) content (**B**) in guava fruit. Standard error showed by vertical bars and each value is mean of three replicates. Different letters on the top of bars indicate significant differences at *p* ≤ 0.05.

An increase in MDA content is associated with membrane injury in fruit, resulting in membrane rupture or shriveling of the fruit’s skin. Lipids in the epidermis are oxidized into MDA, which is indicative of damage to the fruit membrane [27]. The MDA content was assayed in control and treated samples under storage (12 days). The application of AV gel coatings (20, 40, 60, and 80%) suppressed MDA production in comparison to control samples (Figure 4A). Overall, MDA content increased gradually in treated fruits, whereas rapid increases were measured in control samples. It was evident that the 60% and 80% AV gel treatments were more effective in reducing the levels of MDA and in inhibiting the shriveling of the fruits. Our results are also consistent with the effects of chitosan coating on guava fruits during cold storage [7].

### 3.5. TPC

The highest phenolic content was observed at the beginning of the storage period, and no change was found in control fruits up to the third day. However, later, an abrupt decrease in TPC was observed (Figure 4B) in the untreated fruits. In contrast to the control samples, the AV gel-treated samples maintained their phenolic content. Phenolic compounds are secondary metabolites that possess antioxidant activity, which can inhibit free radical formation during oxidative stress. TPC retard the oxidative degradation of lipids and maintain the nutritional value of food [43]. These results are consistent with results reported by researchers who worked extensively in the area of phenolic content in fruits during storage and ripening [44]. Similar results were reported in gabiroba fruit grown in Brazil and stored at different temperatures [45]. Furthermore, a similar decrease in phenolic content was been reported in litchi fruit [25].

### 3.6. Carotenoids, TAC, and AsA

**Figure 5:**
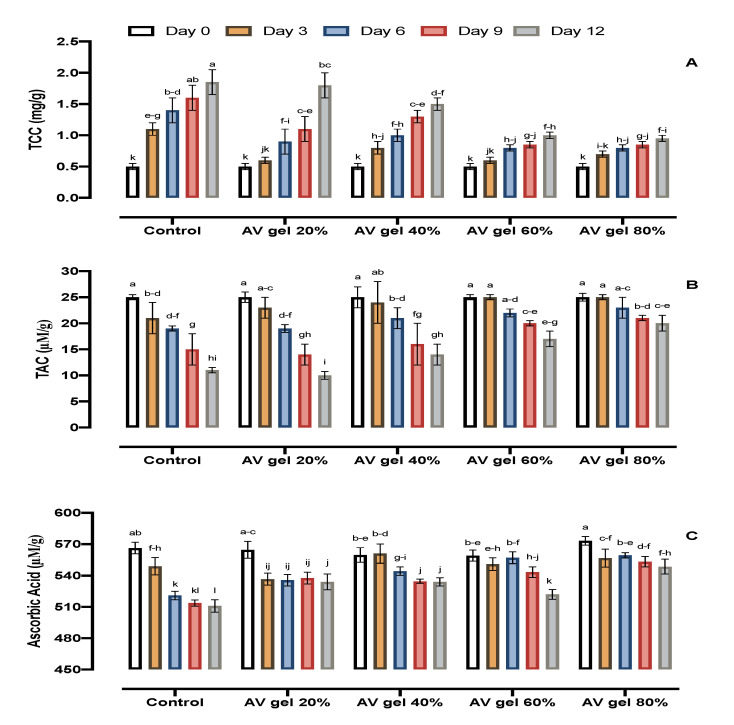
Effects of AV gel coating on total carotenoids (**A**), total antioxidant capacity (TAC) (**B**), and ascorbic acid (**C**) in guava fruit. Standard error shown by vertical bars; each value is the mean of three replicates. Different letters on the top of the bars indicate significant differences (*p* ≤ 0.05).

Total carotenoid contents increased significantly in untreated control samples, whereas AV gel-treated samples exhibited smaller increases in total carotenoids during storage (Figure 5A). Oxidation (the main cause of losses of carotenoids) depends on oxygen availability and is promoted by metals, light, co-oxidation. In guava fruits, it is vital to maintain a high carotenoid content, as these molecules are dietary bioactive compounds that provide defense to counter many degenerative conditions [46]. These results are in agreement with the results of authors who found that carotenoid concentration increases during fruit maturation and ripening [47].

TAC is the activity of total antioxidants against oxidative stress. We observed that TAC progressively decreased in AV gel-treated and control samples during the entire period of storage (Figure 5B). AV gel treatment slowed down the reduction of TAC compared to control fruits after 12 days of storage. AV gel-treated fruits had a greater TAC than control fruits after 12 days of storage. The results in the present study are in accordance with those of previous studies, indicating that a higher TAC of guava fruit is considered suitable to alleviate oxidative stress [34]. Generally, as the production of free radicals increases, TAC declines in guava tissues during prolonged storage [43]. Potentially, AV gel coating has a good ability to preserve the TAC because it reduces free radical production [48]. Hence, AV gel treatment likely maintained TAC due to reduced senescence and lower production of H_2_O_2_ and O_2_.

Ascorbic acid plays a role as an antioxidant as it helps to scavenge free radicals and, in that way, prevents the deterioration of fruit during ripening, owing to oxidation. The AsA content in control samples decreased significantly during storage, and this decline was also observed in AV-treated guavas. No significant difference in AsA concentration was observed in AV gel-treated and control guavas from the third to the nineth day of storage (Figure 5C). However, with further progression of the storage period, the AsA content was sharply reduced, though AV gel-treated fruit still maintained higher values of AsA than the control samples. The AsA content decreases during storage due to its oxidative breakdown [49]. AV gel coating inhibits oxidation, which limits the reduction of AsA during the postharvest period. AV gel coating restricts O_2_ availability for oxidative breakdown, thereby reducing fruit degradation and fruit senescence [50]. AV gel coating solutions reduced AsA oxidation; hence, ascorbic acid content was higher in all AV gel-coated fruits, in particular fruits treated with the 80% AV gel. These findings are in accordance with those reported for tomatoes coated with an aloe vera gel, for which it was observed that the gel delayed the decline of ascorbic acid during storage [51].

### 3.7. TFC

**Figure 6:**
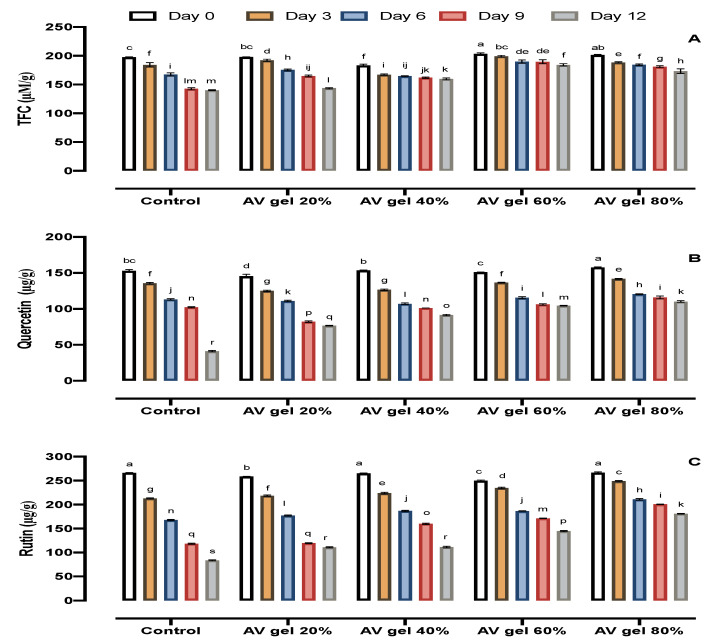
Effects of AV gel coating on total flavonoids content (TFC) (**A**), quercetin (**B**), and rutin (**C**) in guava fruit. Standard error shown by vertical bars; each value is the mean of three replicates. Different letters on the top of the bars indicate significant differences (*p* ≤ 0.05).

Total flavonoid content decreased during storage in the control fruits. This reduction in TFC was retarded by AV gel treatment in a dose-dependent manner (Figure 6A). The AV gel-treated fruits showed a 1.65-fold higher TFC in contrast to the control samples. In 2018, researchers observed higher TFC in guava fruits coated with chitosan and pomegranate peel extracts [28]. Flavonoids have antioxidant activity with positive effects on the shelf life of fruits. Phenylalanine ammonia lyase (PAL) is the main enzyme in the synthesis of phenolics and flavonoids in plants. It was reported that PAL activity increased in raspberry fruit treated with AV gel coatings during storage [48].

### 3.8. Quercetin and Rutin

The quercetin and rutin contents decreased throughout the storage period regardless of the treatment, but it was observed that in AV gel-treated fruits their decline was comparatively slower than in control fruits (Figure 6B,C). The decrease in quercetin and rutin was observed from the start to the end of the storage period and ranged from 154.5 µg/g to 42.17 µg/g and from 267.10 µg/g to 117.10 µg/g, respectively, in control fruits. In fruits treated with AV gel 80%, quercetin and rutin concentrations declined from 158.43 µg/g to 111.32 µg/g and from 268.12 µg/g to 181.83 µg/g, respectively, during storage. AV gel 40% and AV gel 60% treatments also attenuated the losses of quercetin and rutin throughout the postharvest storage period. Quercetin and rutin are used as ingredients in various multivitamin supplements [52]. Phenolic and flavonoid compounds act as primary antioxidants [53] and are known to react with hydroxyl radicals and superoxide anion radicals [54]. AV gel facilitates the induction of phenylalanine ammonia lyase biosynthesis by flavonoids in fruit, which might be responsible for maintaining a high flavonoid content for a longer period of time [55]. These outcomes are in close agreement with those of other authors who applied different coatings on fruits and found them effective for preserving flavonoids content in tomato [56] and papaya [57]. Our results indicate that AV gels maintained quercetin and rutin contents during the storage period.

### 3.9. APX, CAT, and SOD Activities

**Figure 7:**
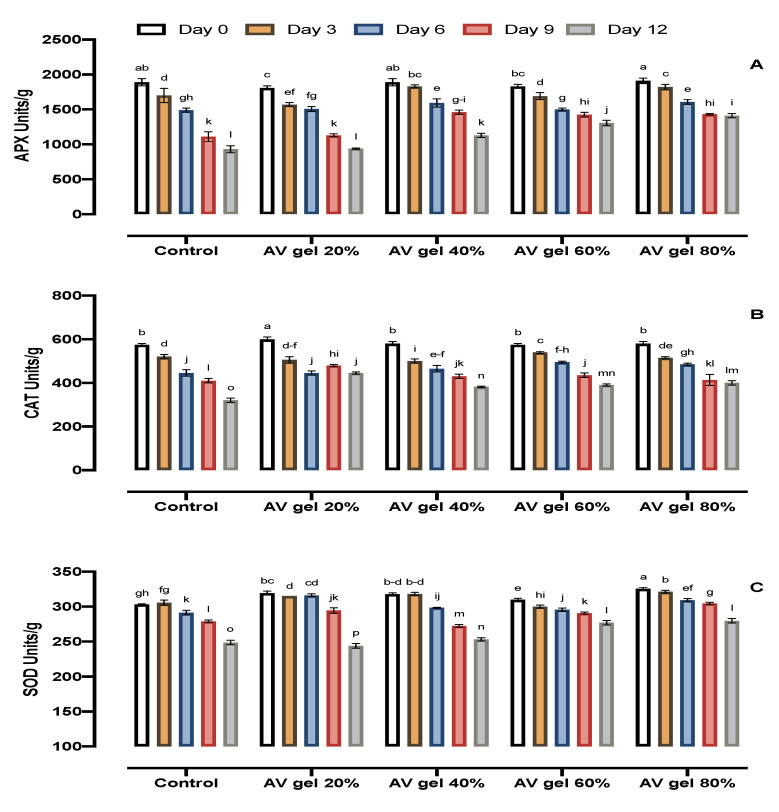
Effects of AV gel coating on ascorbate peroxidase (APX) (**A**), catalase (CAT) (**B**), and superoxide dismutase (SOD) (**C**) in guava fruit. Standard error shown by vertical bars; each value is the mean of three replicates. Different letters on the top of the bars indicate significant differences (*p* ≤ 0.05).

APX enzyme activity in AV gel-treated samples decreased from the third day to the nineth day during storage, whereas, in control fruits, the activity declined at a noticeable rate throughout the storage period (Figure 7A). Moreover, CAT enzyme activity also showed a continuous reduction in all experimental conditions. However, the rate of CAT activity decrease was greater in untreated fruits than in fruits treated with AV gels (Figure 7B). The activity of SOD enzyme also reduced progressively during storage, although SOD activity losses were smaller in treated fruits than in control fruits (Figure 7C). AV gel coating can decline the senescence rate and maintain the activity of antioxidant enzymes [58]. To control the reactive oxygen species (ROS) levels and to protect cells under stress conditions, tissues contain enzymes for scavenging ROS. ROS in plant tissues could be scavenged by antioxidant enzymes preventing the harmful effects of H_2_O_2_ in tissues [7]. In our experiment, fruits coated with 80% and 60% AV gels had higher enzymatic activities than control fruits, indicating that AV gel coating delayed the senescence of guava fruits. The antioxidative enzymes SOD, CAT, and APX detoxify different free radicals and reduce browning by alleviating oxidative damage. Therefore, maintaining higher activities of the said enzymes is imperative to reduce the incidence of browning [59].

## 4. Conclusions

The results of this research demonstrate that chemical-free edible coatings like AV gels have potential to prolong the marketability of guava fruit. AV gel coating was found to be effective in maintaining the postharvest quality of guava fruit. Treatments with the AV gels prevented storage-induced changes such as weight loss, changes in total soluble solids and total chlorophyll content, while maintaining the contents of sugars. In general, the 80% AV gel treatment proved to be the most effective in improving the postharvest life, as evidenced by the values of the parameters assessed in this research. AV gel treatment reduced lipid peroxidation, consistent with its maintenance of antioxidants, i.e., TPC, TAC, TF, AsA, and carotenoids during the storage period. AV gel coating also prevented the decline in enzymatic antioxidants including APX, CAT, and SOD. AV gel coating can be considered an effective and environment friendly, non-chemical treatment for delaying ripening processes and retaining guava fruit quality through redox homeostasis during postharvest storage.
